# Educating emergency department nurses about trauma informed care for people presenting with mental health crisis: a pilot study

**DOI:** 10.1186/s12912-016-0141-y

**Published:** 2016-03-24

**Authors:** Andrea Hall, Brian McKenna, Vikki Dearie, Tessa Maguire, Rosemary Charleston, Trentham Furness

**Affiliations:** Emergency Department, The Royal Melbourne Hospital, Melbourne Health, Parkville, Australia; School of Clinical Sciences, Auckland University of Technology, Auckland, New Zealand; Western Victorian Mental Health Learning & Development Cluster, Melbourne Health, Parkville, Australia; Forensicare, Fairfield, Australia; Centre for Forensic Behavioural Science, Swinburne University, Clifton Hill, Australia; School of Nursing, Midwifery and Paramedicine, Australian Catholic University, Fitzroy, Australia; NorthWestern Mental Health, The Royal Melbourne Hospital, Parkville, Australia

**Keywords:** Trauma informed care, Emergency department, Nurses, Mental illness

## Abstract

**Background:**

Practicing with trauma informed care (TIC) can strengthen nurses’ knowledge about the association of past trauma and the impact of trauma on the patient’s current mental illness. An aim of TIC is to avoid potentially re-traumatising a patient during their episode of care. A TIC education package can provide nurses with content that describes the interplay of neurological, biological, psychological, and social effects of trauma that may reduce the likelihood of re-traumatisation. Although mental health nurses can be TIC leads in multidisciplinary environments, the translation of TIC into clinical practice by nurses working in emergency departments (EDs) is unknown. However, before ED nurses can begin to practice TIC, they must first be provided with meaningful and specific education about TIC. Therefore, the aims of this study were to; (1) evaluate the effectiveness of TIC education for ED nursing staff and (2) describe subsequent clinical practice that was trauma informed.

**Methods:**

This project was conducted as exploratory research with a mixed methods design. Quantitative data were collected with an 18-item pre-education and post-education questionnaire. Qualitative data were collected with two one-off focus groups conducted at least three-months after the TIC education. Two EDs were involved in the study.

**Results:**

A total of 34 ED nurses participated in the TIC education and 14 ED nurses participated in the focus groups. There was meaningful change (*p* < 0.01, *r* ≥ 0.35) in 9 of the 18-items after TIC education. Two themes, each with two sub-themes, were evident in the data. The themes were based on the perceived effectiveness of TIC education and the subsequent changes in clinical practice in the period after TIC education.

**Conclusion:**

Emergency department nurses became more informed of the interplay of trauma on an individual’s mental health. However, providing care with a TIC framework in an ED setting was a considerable challenge primarily due to time constraints relative to the day-to-day ED environment and rapid turnover of patients with potentially multiple and complex presentations. Despite this, nurses understood the effect of TIC to reduce the likelihood of re-traumatisation and expressed a desire to use a TIC framework.

## Background

Practicing with trauma informed care (TIC) can strengthen nurses’ knowledge about the association of past trauma and the impact of trauma on the patient’s current mental illness [1]. An aim of practicing within a TIC framework is to avoid potentially re-traumatising a consumer during an episode of care in hospitals or other clinical settings [2]. Re-traumatisation may evoke memories of violence and victimization [3] and be symptomatic of, for example, posttraumatic stress disorder [4].

The emphasis on practicing within a TIC framework has occurred in mental health settings. Hence, a TIC education package can provide mental health nurses with content that describes the interplay of neurological, biological, psychological, and social effects of trauma on an individual’s mental health [5]. Such education may reduce the likelihood of re-traumatisation that may escalate to aggressive and violent behaviour [6, 7], risk of harm to self and others [8] and the subsequent use of restrictive interventions [1]. Restrictive interventions may include the use of seclusion, physical restraint, or mechanical restraint and are known to exacerbate symptoms of past trauma for people with mental illness [9–11]. Nurses practicing within a TIC framework have reduced the use of restrictive interventions within inpatient settings [12–14]. However, mental health nurses may struggle to translate the values of TIC into their every-day clinical practice due to factors such as practicing within risk adverse, punitive, and austere clinical settings [1].

Episodes of seclusion [15, 16] physical restraint [17–19], and mechanical restraint [20] may also occur in hospital EDs when people present in mental health crisis and are associated with adverse psychological outcomes for those patients affected [20, 21]. In Victoria, restrictive interventions occur despite a policy directive that all health services are required to reduce the use of restrictive interventions with a goal of eliminating the practices [22]. Additionally, Victorian EDs have been provided with a framework to reduce restrictive interventions [22], which includes the use of TIC, yet the challenge remains to educate ED nurses about its use and embed such knowledge and skills into practice.

Although mental health nurses can be TIC leads in multidisciplinary environments [2], the translation of TIC into clinical practice for nurses working in EDs is unknown. Therefore, before ED nurses can begin to use TIC in an attempt to reduce the use of restrictive interventions, they must first be provided with meaningful and specific education about the interplay of neurological, biological, psychological, and social effects of trauma on an individual’s mental health. Furthermore, ED nurses also need the opportunity to experience and practice acquired knowledge in supportive peer environments. Such processes may enhance ED nurses’ skills to potentially reduce the likelihood of re-traumatisation of patients presenting to an ED and reduce the subsequent need for the use of restrictive interventions. Therefore, the aims of this study were to; (1) evaluate the effectiveness of a tailored TIC education package for ED nursing staff and (2) describe subsequent clinical practice that was trauma informed after TIC education.

## Methods

### Study design

This project was conducted as exploratory research with a mixed methods design to meet the aims. Mixed methods research designs attempt to combine both the methodology and philosophy of quantitative and qualitative research [23]. Quantitative data were collected with a pre-education and post-education questionnaire on the day of the TIC education. Qualitative data were collected with two one-off focus groups conducted at least three months after the TIC education package was introduced at the service. One urban and one rural ED were involved in the study. The EDs were selected based on; (1) the capacity to concurrently treat paediatric and adult patients, (2) the potential to admit those patients to an acute mental health inpatient unit at the hospital campus, and (3) the ability to meet the research design of the study. Data were collected from November 2014 through February 2015. This study was approved by the Human Research Ethics Committee of the Frankston Hospital (LRR/14/PH/36) and the Bendigo Hospital (LNR/15/BHCG/13).

### Participants

The Nurse Unit Manager and/or the ED Educator at each ED assisted in distributing invitations to participate via email and flyer to all currently employed ED nurses at each campus. The email and flyer outlined the aims of the study and included the contact details of the research team to enable potential participants to contact the researchers directly to arrange voluntary participation in the TIC education and focus group.

### Study protocol

To meet the aims of the study, participants completed; (1) a pre-education questionnaire, (2) a single day TIC education package, (3) a post-education questionnaire, and (4) a three-month follow-up focus group.

In 2013, the Victorian Government Department of Health released a ‘Framework for Reducing Restrictive Interventions’ [22]. The framework was created with people with lived experience of mental illness and other key stakeholders and supported with a ‘Reducing Restrictive Interventions literature review and document analysis’ [24]. A focus of the framework was to describe how TIC can reduce the use of restrictive interventions. Subsequently, a TIC training package was developed by the Victorian Reducing Restrictive Interventions Project Team [25]. The multidisciplinary Project Team were funded/employed by the Victorian Department of Health based on expertise in mental health or ED settings, or lived experience of mental illness. An aim of the Project Team was to deliver the TIC training package in an attempt to educate senior ED nurses about TIC and as a consequence allow those ED nurses to train their colleagues with a foundation of TIC. The content of the package focused on understanding TIC and the confidence to practice TIC. The educational package was co-produced and co-delivered by the Project Team consisting of an emergency nurse, mental health nurses, a person with lived experience of mental illness, a carer consultant, and supported with a training manual and visual presentation materials.

The package was delivered on a single day and was modularised to enable short bursts of education/training (see Table 1). There were eight 45 minute modules: An introduction to TIC; the neurobiological impacts of trauma; the social consequences of trauma; a theoretical framework on the impact of trauma; an exploration of the extent to which service delivery can impact on past trauma experiences; communication skills in relating to trauma; the impact of TIC on the clinical workforce; and developing a clinical commitment to TIC following the training.

**Table 1:** Content of the TIC Modules

Module	Topic	Objectives
1	Introduction to Trauma Informed Care	• Have a basic introductory level understanding of what TIC is
		• Be aware of the prevalence of complex trauma in the mental health population and its consequences
2	Neurobiology	• Display an introductory level understanding on the neurobiological consequences of trauma
		• Describe the neuro-hormonal changes as a result of trauma
		• Be able to articulate the effect of trauma on brain development, including the neuro-sequential model
3	Social Consequences of trauma	• Discuss social consequences of prolonged trauma
		• Have knowledge of the Adverse Childhood Experiences study
		• Have an understanding of the various types of childhood trauma
		• Have an introductory level understanding of the social consequences of trauma
4	The Cognitive Model of Trauma	• Describe the Cognitive Model of Trauma
		• Discuss how beliefs formed in childhood form the basis of much of the behaviour we see in patients
		• Explain how short-term solutions become long-term problems
5	“The self-fulfilling prophecy”	• Describe parts of Young’s schema processes model
		• Explain how some services/processes reinforce negative beliefs
		• Discuss ways to avoid reinforcing negative beliefs
6	Responding to stories	• Describe 2 ways of discussing traumatic memory
		• Discuss staff concerns and solutions around talking about trauma
		•Describe Davidson’s “compassion narrative”
7	Trauma and the Workforce	• Discuss the effects of stress on mental health professionals
		• Have knowledge of strategies to improve self-care
		• Examine positive reasons for working in mental health
8	Where to from here	• Discuss how the training has influenced knowledge of trauma and its effects
		• Discuss how to begin implementing this knowledge in the workplace
		• Describe one change which can be made with immediate effect

The package was based on adult learning principles that participants offer experiences that contribute to the learning of others [26]. A variety of modes of presentation were adopted to accommodate the differing learning styles of participants including slide show presentations, video viewing, group discussion, and interactive learning ‘games’. The co-facilitation occurred with a person with lived experience of mental illness and trauma, who at times reflected on their own experiences, and all other members of the Project Team.

### Measurements

Quantitative data: Staff completed a pre-education and post-education 18-item questionnaire derived from the content of the eight modules. For example, ED nurses were asked to rate their level of confidence to respond to patients’ disclosures of trauma and understanding if their current nursing practice is trauma informed (see Table 3 in results). The questionnaire was completed immediately before and after ED nurses had participated in the education. Data were collected on a 5-point Likert scale [27], where a rating of 1 represented strong disagreement and a rating of 5 represented strong agreement.

Qualitative data: A total of two 40-minute focus groups were completed (one at each ED) at least three-months after completion of the TIC education. The focus groups were constructed to explore staff perceptions of experiences and benefits of TIC in the ED. For example, participants were asked to discuss “what changes have you made in your everyday work since the TIC education” and “how have you applied what you have learned into your practice, with concrete examples.” The 13-item focus group interview schedule was identical across the two focus groups and conducted by the same researchers (AH and/or VD). Data were recorded using an audio-digital recorder (Sony ICD-PX333M) and field notes were integrated into the data analysis.

### Statistical analyses

For each of the 18-items of the questionnaire, datum was collated and change computed with Wilcoxon matched tests in the Statistical Package for Social Scientists 21.0 for Windows (IBM Corp, Armonk, USA). Significance was accepted at *p* ≤ 0.001 with Bonferroni corrections. Effect size (*r*) was computed to describe the magnitude of the change in ratings where 0.1 was considered small, 0.3 moderate, and 0.5 large effect [28].

A thematic analysis of the focus groups was undertaken using a general inductive approach. This approach allows defensible analysis of qualitative data that may initially be varied raw text and allows it to be condensed into brief summaries [29]. Data were analysed with the processes of immersion, coding, creating categories, and identifying themes [30]. Interview data were transcribed verbatim, integrated with field notes and independently read repeatedly by two researchers (BM & TM). Colour codes were developed through agreement among the researchers and categories were formed. Categories were collapsed to themes and examined for supporting quotes from the data [31, 32].

## Results

A total of 34 nurses provided informed voluntary consent to complete the pre- and post-education questionnaire. The majority of participants were between 31 and 40 years of age and had been working in an ED between one and 10 years (see Table 2). A small number of participants had previous experience working as a mental health nurse in a mental health setting.

**Table 2:** Descriptive statistics of ED nurses enrolled in TIC education

Variable	Description	Count (*n*)^a^	%	Missing data (*n*)
Age in years	20–30	8	24	0
	31–40	13	38	
	41–50	8	24	
	> 50	5	14	
Gender	Female	22	71	3
	Male	9	29	
Qualification	Hospital Trained	1	3	2
	Diploma of Nursing	4	13	
	Undergraduate Degree	7	22	
	Postgraduate Certificate	17	53	
	Postgraduate Diploma	2	6	
	Postgraduate Masters	1	3	
Years employed as a Nurse	1–5	4	12	1
	5–10	10	30	
	11–15	10	30	
	16–20	1	4	
	> 20	8	24	
Years employed in an ED	1–5	16	50	2
	5–10	9	28	
	11–15	3	10	
	16–20	2	6	
	> 20	2	6	
History of working in a mental health setting	Yes	4	12	1
	No	29	88	

^a^ *N* = 34 (Rural nurses *n* = 16, Urban nurses *n* = 18)

### Quantitative findings

After TIC education, ED nurses reported more confidence in their ability to, talk to patients about traumatic experiences (*p* = 0.001, *r* = 0.41), respond to disclosures of family violence (*p* = 0.001, *r* = 0.41), and understand how their current nursing practice is trauma informed (*p* = 0.001, *r* = 0.53) (see Table 3). However there was no effect of the TIC education on the understanding that it is the ED nurses role to listen to patients’ talk about their trauma (*p* = 0.171, *r* = 0.17), comprehension of the contribution of the ED environment to trauma (*p* = 0.209, *r* = 0.15), or feeling confident about how to respond to patients' disclosure about trauma (*p* = 0.188, *r* = 0.16).

**Table 3:** Effects of TIC education for ED nurses

Questions	Pre	Post	*z*-score	*p*-value	*r*-value
I am confident talking with patients about their traumatic experiences	3.2	3.9	−3.333	0.001*	0.41
Childhood trauma is likely to have an impact on a person’s mental health	4.4	4.8	−2.351	0.019	0.29
Most patients who access the ED have experienced interpersonal trauma	3.5	4.0	−2.275	0.023	0.28
I feel confident to respond when a patients tells me he/she is currently experiencing family violence	3.3	3.9	−3.331	0.001*	0.41
I feel confident supporting patients to talk about what they feel comfortable to disclose about their previous trauma	3.5	4.2	−3.038	0.002	0.37
The physical environment of the ED can contribute to people feeling unsafe	4.3	4.6	−1.258	0.209	0.15
I do not feel confident recognizing when someone is re-experiencing a traumatic event	3.3	2.4^a^	−2.851	0.004	0.35
It is not part of my role to listen to patients’ talk about their trauma	1.9	1.5^a^	−1.369	0.171	0.17
My current nursing practice is trauma informed	2.7	3.9	−4.279	0.001*	0.53
I feel confident acknowledging how hard it must be to talk about trauma	3.7	4.2	−1.810	0.070	0.22
I have a good understanding about what TIC means	2.7	4.2	−4.814	0.001*	0.59
Responding to previous trauma is not part of my role	2.2	1.8^a^	−1.284	0.199	0.16
There is a strong link between childhood trauma and brain development	3.8	4.3	−1.997	0.046	0.25
I feel confident talking with patients about their coping strategies to deal with the impact of trauma	2.9	3.9	−3.984	0.001*	0.49
I do not know how to ask questions about childhood trauma	3.4	2.4	−3.094	0.002	0.38
I feel confident about how to respond to patients’ disclosure about trauma	3.4	3.7	−1.317	0.188	0.16
I know which colleague I can talk to about trauma issues when I feel uncertain as to how to respond	3.8	4.2	−1.795	0.073	0.22
I can explain to patients what trauma is, including its effects	2.8	3.8	−4.123	0.001*	0.51

Data are mean. *N* = 33 as post-data missing for one nurse

*p ≤ 0.001 with Bonferroni correction

^a^Reverse coded

### Qualitative findings

Of the 34 ED nurses that completed the TIC package, 14 participated in the focus groups (seven from each ED). Two themes, each with two sub-themes, were evident in the data (see Table 4). The themes were based about the perceived effectiveness of TIC education and the subsequent changes in clinical practice in the period after TIC education.

**Table 4:** Themes and sub-themes of the qualitative analysis

Theme	Sub-theme
Effectiveness of the TIC education	• Improved understanding of TIC
	• Beginnings of an attitudinal change
Changes in nursing practice	• Improvements in a person-centred approach
	• Limitations of TIC

### Effectiveness of the TIC education

#### Improved understanding of TIC

After the TIC education, some participants were able to discuss TIC without prompts, including a very specific understanding of TIC which incorporated past experiences impacting on current presentation. Participants were also cognizant of the effects of the use of restrictive interventions and subsequent re-traumatisation. Participants attributed this improved understanding to specific aspects of the TIC education. The input of a consumer consultant, who was a co-facilitator and discussed the lived experience of trauma, was highlighted as being both valuable and memorable in this regard:

*“That day, everything we learnt that day, that consumer participant. She’s the one who has stayed in my mind so when I do see someone who I think is just going off she’s the face that comes back so that firsthand really, really works as far as education goes because that woman, she went through a terrible lot and a lot of that seemed preventable I thought.”* (Focus Group 1)

Many of the participants found the neurobiology component of the education assisted their understanding of trauma and the impact this can have on a person:

*“… with trauma informed training and education you get to understand, particularly for the nurses, that through someone’s growth and development their brain may not have developed the same as someone who hasn’t had those traumas, so it’s almost something that physically they can’t help. So to be able to put the face to the story to what’s physically going on probably just connects it really, really well.”* (Focus Group 1)

The participants agreed they would benefit from more TIC education. Furthermore, the consensus was that the TIC education could benefit other ED staff (i.e., medical and auxiliary staff) and emergency services staff (i.e., ambulance and police officers) in an attempt to avoid the potential for behavioural escalation and the subsequent use of restrictive interventions.

#### Beginnings of an attitudinal change

The benefits of the TIC education were not merely limited to a cerebral exercise involving knowledge and skill acquisition. Nurses’ improved understanding of TIC translated into attitudinal shifts for some, but not all participants. As avidly described by one participant:

*“I went into the day thinking ‘oh this is all mamby pamby …. to be perfectly frank. I came out actually with a much better attitude … I think it was certainly valuable.”* (Focus Group 1)

For some participants, the visceral connection to attitude enabled a reflection on deep-seated views explaining the behaviour of some people presenting to the ED:

*“Previously I’d always seen challenging behaviours as annoying in the emergency department…but the biggest thing I took* [from the education] *was that there’s a reason for that, there’s always something underlying. But I don’t think cognitively I actually considered that before doing the trauma informed day.”* (Focus Group 2)

### Changes in nursing practice

#### Improvements in a person-centred approach

Although there was no effect (*p* = 0.199, *r* = 0.16) of the TIC education on the understanding that it is the ED nurses role to respond to a patient’s previous trauma, some nurses discussed their increased openness to ask questions about trauma and listen to the patient’s responses. Such realisations enabled an enhanced person-centred care approach. There was also an increased emphasis on communication including providing reassurance:

*“I think for me it’s just communication and just engaging the person as much as possible in conversations.... reassuring them that they’re safe as much as possible.”* (Focus Group 1)

Having an understanding about the impact of trauma and the importance of reassuring patients enabled a more holistic understanding of the patient and their experience. Participants independently discussed instances of the use of restrictive interventions despite the TIC education. However, there was an indication that the TIC education was impacting on reducing the use of restrictive interventions, through a person-centred approach.

*“The person may have still needed to be restrained but we were able to use less restraint and I think a lot of that was through actually engaging the person in the whole process and getting them to work with us rather than seeing it as we were sort of doing things against them. So we were trying to work with them as much as possible as well.”* (Focus Group 1)

#### Limitations of TIC

Some of the participants spoke of the environmental complexities and pressures of the ED on their ability to use TIC as a framework to reduce restrictive interventions. Although participants described the intent to use TIC as a framework, some misgivings were expressed about the ability to implement the initiatives within an ED setting. There were two primary limitations in implementing TIC. The first was time constraints relative to the general day-to-day requirements of providing care in an ED characterised with rapid turnover and complex presentations. The other was perceived risk of harm to staff, from those people presenting to the ED acutely aroused and exhibiting aggressive and violent behaviour.

## Discussion

People presenting to an ED with acute mental health crisis may have experienced a traumatic event such as interpersonal violence or severe neglect in the days or years preceding the ED attendance [5]. As such, methods of practice by ED nurses from a TIC framework may reduce the likelihood of re-traumatisation, yet little is known of the effect of practicing TIC in ED settings. Within mental health inpatient settings, TIC has been an effective strategy to reduce the likelihood of re-traumatising patients and reducing the use of restrictive interventions [14]. The results of the current study identified that ED nurses can become more informed of the interplay of neurological, biological, psychological, and social effects of trauma on an individual’s mental health. Such knowledge transfer may assist ED nurses to practice using TIC principles. However, prior to such, a shift in clinical mindfulness, attitudes about the usefulness of TIC, and pragmatic application of TIC in an ED setting require further development.

Participants in the current study were; (1) unsure if responding to past trauma was a component of an ED nurse’s role, (2) unsure if the ED environment contributes to trauma, (3) unsure about which colleagues may assist in working with patients who have a past history of trauma, and (4) unsure how to respond to patient’s disclosures of past trauma. Although the participants gained understanding of some of the theory of TIC (i.e., modules 1, 2, 4, 5), it was clear that the practical application of TIC (i.e., modules 3, 6, 7, 8) need developmental consideration. The findings from this study support similar concerns expressed by mental health nurses who are unsure about what actions they can take to support TIC practice [1].

A review by Muskett (2014) identified that successful integration of TIC needs active leadership, mentoring, regular debriefing, ongoing TIC education, and involvement of consumers and carers [1]. However, the review is specific to mental health inpatient services. As such, translation of knowledge to practice may be challenging due to the different organisational procedures among the two settings. The results of the current study identified the lack of required support to integrate and maintain a focus of TIC in ED settings with particular limitations on time and difficulties regarding managing aggressive behaviour exhibited by some people presenting in acute medical and/or mental health crisis. However, processes that encourage reflection on practice may assist in embedding the knowledge translation. Action Learning Sets, for example, where clinicians learn how to ask questions of colleagues, rather than find answers to questions [33] may benefit collegial learning about how to practice with TIC in ED settings. Furthermore, the involvement of the person with lived experience of mental illness in the current study was viewed positively. Providing a resource for the ongoing supportive role of this expertise for staff in an ED may assist in the translation of knowledge into practice.

The caveat remains however, that mental health presentations to EDs are increasing [34] as is the risk associated with aggressive and violent behaviour [35] and the subsequent use of restrictive interventions [36]. Such challenges require genuine embedding of knowledge into practice which requires attitudinal change. On-going attention is required to determine how such change is best supported. Once the attitude shift has occurred and TIC practice embedded, an evaluation of the impact on the use of TIC to reduce the risk of re-traumatisation and the use of restrictive interventions in ED settings is required.

### Limitations

The current study was limited to a pilot of two EDs. There was no comparison among the urban and rural ED, so data are relative to ED nurses opinions of TIC education and their ability to practice TIC regardless of the geographical setting or profile of patients systemic to those areas. Furthermore, the effect of the TIC education on reducing instances of re-traumatisation and the use of restrictive interventions remain unknown. Although identified by participants that TIC education may benefit other ED staff and axillary services, the effects of such were beyond the scope of the current study.

## Conclusions

The results of the current study identified that ED nurses can become more informed of the interplay of neurological, biological, psychological, and social effects of trauma on an individual’s mental health. However, providing care within a TIC framework in an ED setting was a considerable challenge primarily due to time constraints relative to the day-to-day ED environment and rapid turnover of patients with potentially multiple and complex presentations. Despite the challenging ED environment, nurses understood the effect of TIC to reduce the likelihood of re-traumatisation and described a desire to use a TIC framework in their clinical setting.

## Abbreviations

ED: Emergency Department
TIC: Trauma Informed Care
